# Reviewed article: Farias AF, Gava ML, Vieira SKMF, Araújo GS, Mangabeira CL, Traebert E, Traebert J, Trevisol DJ, Schuelter-Trevisol F. Prevalence of sexually transmitted infections among incarcerated men in Prison Complex: a cross-sectional study, Santa Catarina, 2023-2024. Epidemiol Serv Saude. 2026:35;e20250140

**DOI:** 10.1590/S2237-96222026v35e20250140.a

**Published:** 2026-02-16

**Authors:** Ricardo Burg Ceccim

**Affiliations:** 1 Universidade Federal do Rio Grande do Sul, Porto Alegre, RS, Brazil Universidade Federal do Rio Grande do Sul Porto Alegre RS Brazil

## Peer review A

## Questionnaire

Does the manuscript contain new and significant information to justify publication? Yes

Does the Abstract (Summary) clearly and accurately describe the content of the article? Yes

Is the problem significant and concisely stated? Yes

Are the methods described comprehensively? Yes

Are the interpretations and conclusions justified by the results? Yes

Is adequate reference made to other work in the field? Yes

## Manuscript Structure

Length of article is: Adequate

Number of tables is: Adequate

Number of figures is: Adequate

Please state any conflict(s) of interest that you have in relation to the review of this paper. None

Interest: Excellent

Quality: Good

Originality: Average

Overall: Average

Recommendation: Minor Revision

## Comments

O texto traz dados que sempre devem ser atualizados para subsidiar as políticas, então é bem-vindo a uma revista de epidemiologia em serviços de saúde. Os dados sugerem subsídio apara pelo menos três políticas públicas: políticas intersetoriais destinadas à juventude (população dos 18 aos 29 anos), política de saúde do homem (em especial de HSM sem o uso de preservativo) e política de saúde para a população prisional. Deve-se apontar, contudo, que alguns dados não podem ser discutidos simplesmente por expressão de maioria, sob o risco de obnubilar informação necessária. Por exemplo o tema da raça/cor, pois a distribuição entre brancos e negros/pardos é praticamente 50% para cada grupo, sendo a sífilis predominante no gripo da raça/cor negra/parda. A faixa etária da juventude deve ser destacada do agrupamento 18-39 anos de idade, pois a faixa etária 18-29 anos de idade é alvo das políticas intersetoriais da juventude, sendo uma faixa diante da qual estudos de exposição e vulnerabilização devem indicar ações de promoção e proteção da saúde específicas.

Quanto ao ensino, se 64% da população carcerária masculina é do ensino fundamental completo ou incompleto, 46% estão entre ensino médio incompleto e superior incompleto, pode ser pensada em programas de comunicação, informação e promoção da saúde. A maior parte da população carcerária do estudo é heterossexual, o que também é um dado sobre masculinidade e cuidado com a saúde e o corpo, assim sobre comportamento de gênero. O número de parceiros sexuais dessa população não diverge da população em geral, não havendo particular produção de evidência, além daquelas de atenção à saúde da população gay e bissexual. A prevalência mais alta das IST na população privada de liberdade em relação à população em geral é coerente com o perfil da população mais exposta e a população carcerária (baixa escolaridade, baixa renda, de periferia, menor acesso aos bens culturais, educacionais e sanitários, menor frequência aos serviços de saúde etc.). Sugere-se rever o artigo sob a luz dessas problematizações, tendo em vista contribuir com o cuidado integral e cidadão das pessoas privadas de liberdade em diferentes políticas públicas.

